# Forecasting elections with agent-based modeling: Two live experiments

**DOI:** 10.1371/journal.pone.0270194

**Published:** 2022-06-30

**Authors:** Ming Gao, Zhongyuan Wang, Kai Wang, Chenhui Liu, Shiping Tang

**Affiliations:** 1 Center for Complex Decision Analysis, Fudan University, Shanghai, China; 2 MOE Laboratory for National Development and Intelligent Governance, Fudan University, Shanghai, China; 3 School of International Relation and Public Affairs, Shanghai International Studies University, Shanghai, China; Sichuan University, CHINA

## Abstract

Election forecasting has been traditionally dominated by subjective surveys and polls or methods centered upon them. We have developed a novel platform for forecasting elections based on agent-based modeling (ABM), which is entirely independent from surveys and polls. The platform uses statistical results from objective data along with simulation models to capture how voters have voted in past elections and how they are likely to vote in an upcoming election. We screen for models that can reproduce results that are very close to the actual results of historical elections and then deploy these selected models to forecast an upcoming election with simulations by combining extrapolated data from historical demographic record and more updated data on economic growth, employment, shock events, and other factors. Here, we report the results of two recent experiments of real-time election forecasting: the 2020 general election in Taiwan and six states in the 2020 general election in the United States. Our mostly objective method using ABM may transform how elections are forecasted and studied.

## Introduction

Election forecasting has been traditionally dominated by opinion polls, surveys, “fundamentals” and the three approaches centered upon them, namely, opinion aggregation, structuralist models, and various synthetic methods.

Forecasting with opinion aggregation projects the election results by aggregating the results from individual estimations derived from different sources and data, including voter intention polls, prediction markets, wisdom of crowds, expert surveys, and so forth [1,2]. The basic idea behind this approach is that by pooling results from different projections, one can minimize the errors and biases with different sources and data. Forecasting using massive social-media data, now increasingly popular with the availability of various data science methods, operates with the same intuition [3–6]. Notably, the opinion aggregation approach predicts candidates’ chance of victory but not the share of votes obtained by competing candidates or parties.

Essentially, forecasting using opinion aggregation rests on the idea of a capricious electorate that is susceptible to changes engendered by campaign dynamics. In contrast, political scientists have postulated that voters’ preferences and voting patterns can remain fairly stable over long periods of time [7,8]. As a result, these researchers contend that most elections can be explained and predicted by a set of structural rules of voting behavior. Structuralists thus seek to forecast elections with models that use a limited number of core predictive economic and political indicators such as candidate approval rate, economic growth, unemployment, and international competitiveness [9–12]. Because structuralist models are mostly based on electoral theories with some explanatory power, they are greatly favored by the scholarly community. Due to their reliance on slow-moving macro-level variables, structuralist models can forecast results with a longer lead time than opinion aggregation based on polls and surveys. However, this also means that the structuralist approach is less equipped to deal with events and shocks that are closer to the voting date.

Given that both the opinion aggregation approach and structuralist models have their own strengths and weaknesses, synthetic methods seek to combine a weighted average of the two approaches and beyond. The rationale behind such an approach is that by combining individual-level dynamics and structural-level determinants as well as a variety of other inputs and methods, one may obtain greater forecasting accuracy than either method alone [13–15]. Nevertheless, the results of the synthetic forecasts are often found difficult to explain even if they can correctly call the winner.

A major shortcoming of the above three approaches is that voters are mostly missing in forecasting exercises even though they are the key agents in determining the final outcomes. More concretely, the existing approaches do not employ much data at the level of the individual voters (e.g., gender, age, ethnicity, education, income, religion, occupation, etc.). Rather, they mainly focus on capturing how different voters will vote in response to macro-level variations or shock events. Moreover, all three approaches are keen to forecast the winner instead of the precise share of votes by different parties or candidates.

We have developed a novel platform for forecasting real-world elections that is based on agent-based modeling (ABM). To our best knowledge, we are the first to do so. Although ABM has been employed for simulating political competitions before (e.g., [16]), existing attempts have focused on establishing and identifying key dynamics in electoral competitions rather than forecasting elections in real time.

Our platform forecasts elections by systematically simulating voters’ voting behavior. As a result, we can forecast the share of votes obtained by each candidate or party rather than merely a crude outcome of an election (i.e., which side wins). Moreover, our ABM-based approach can provide the forecasting with a much longer lead time (from several months to a year), well before election polls can generate meaningful predictions (usually quite close to the election date). Finally, our approach allows for gauging how different blocs of voters are likely to vote, integrating forecasting accuracy with model interpretability and thus providing candidates and parties with a better reference point for formulating their campaign strategies and adjusting mobilization practices.

We have been experimenting with our platform for several years. In 2016, we released our first real-time forecast for the general election in Taiwan. Since then, we have released our forecasting results for the 2018 Taiwan local elections, 2018 U.S. senator elections in Missouri and West Virginia, the Taiwan 2020 general election, and the U.S. 2020 general election. All the forecasts were released publicly before the elections. Over the years, we have steadily improved our platform by 1) collecting more historical data, 2) training our models with ever-richer data and systematically testing our approach against two different electoral systems (Taiwan and the United States). Our platform has consistently performed well and often more accurately than opinion polls. In this article, we report the progress with two of our latest live experiments: the Taiwan 2020 general election and the U.S. 2020 general election.

The rest of this article is organized as follows. We first explicate our novel forecasting approach using ABM simulation and its standard operational procedures (SOP). In particular, we highlight how ABM can combine electoral studies by political scientists with computational power to make fairly accurate election forecasts without relying on public opinion polls and social media data. Next, we articulate the key technical details of our platform, followed by presenting the main results of two of our latest live forecasting experiments using the platform. We conclude with a discussion of the advantages and limitations of the ABM simulation approach for forecasting elections, as well as its broader implications and future research possibilities.

## Why ABM for electoral forecasting

ABM is a bottom-up approach for simulating the actions and interactions of agents with a specific characteristic within a system with predefined behavior rules to generate emergent outcomes at the aggregate level [17–19]. Agents in ABM can be individuals, social groups, parties, governments, or any other actors of interest. Agents can be endowed with a variety of attributes, ranging from demographic characteristics to socio-economic status, that may shape their actions. An aggregate-level outcome, often stable, then emerges from repeated simulations. As a result, ABM allows researchers to simulate and analyze the macro-level phenomenon of interest from micro-level dynamics of individual behaviors.

Although the first ABM system can be traced back to Thomas Schelling’s [20, chap. 4) famed segregation model and Robert Axelrod’s [21] equally famed “computer tournament” for cooperation, for a long time, ABM had limited application in social sciences due to the limitation imposed by computational capacity. Since the 2000s, as computers have become more powerful and especially with the coming of cloud computing, ABM begins to find much wider application in social sciences. So far, social scientists have employed ABMs to explore issues such as cooperation, terrorism, drug trafficking, social mobility, political participation, economic dynamics, and other topics [22,23]. In particular, ABM allows scientists to examine whether purported mechanisms really operate in a given system and test “what if” scenarios. To our knowledge, however, few have deployed ABM for forecasting real economic, social, and political outcomes.

We began to design and develop our ABM-based platform for electoral forecasting in 2015. Underpinning our project has been two key ideas. The first is that we can derive some initial voting rules of agents (voters) from historical data and then deploy ABM simulation to select (or screen) for models that can capture how different voters have voted in past elections fairly accurately by matching simulated election results with the actual results of past elections. Second, we can then deploy those selected models to forecast an upcoming election with ABM simulation by combining extrapolated individual-level data from most recent demographic record with updated structural-level data on key predictive variables such as economic growth, unemployment rate, the crime rate, and shock events, etc.

Distinctively, our platform is entirely independent from surveys and polls, which are mostly subjective and even biased [2,24]. Our platform does not rely on social media data either, not simply because they are highly subjective, but also we knew, even before the Cambridge Analytics scandal during the 2016 U.S. election, that social media data can be easily manipulated and hence are often contaminated and corrupted.

Instead, our platform is underpinned by objective data and computer simulation. At the same time, we draw insights from electoral studies. Our platform not only incorporates both individual-level factors and structural-level variables but also simulates how voters have voted in past elections by taking voters as the principal agents who act upon their characteristics and respond to the structural environment. This leads to another key advantage: our platform can shed light on how different blocs of voters have voted and are likely to vote in the future.

## Method

We built our platform with two starting assumptions. First, because voters are the central decision-maker in any election, they are modeled as the principal agents in our ABM simulations. Second, in addition to their individual attributes (e.g., age, gender, income, education, occupation), voters’ electoral decision is also shaped by other driving factors including macro socioeconomic factors, candidates’ attributes, and campaign dynamics. From data on all these factors and historical electoral results, we then derive some initial rules for agents’ voting behavior with simple regressions. Very critically, however, these initial rules do not have to be very accurate in the first place because ABM simulations will subsequently select models that can closely reproduce historical electoral results.

**Fig 1:**
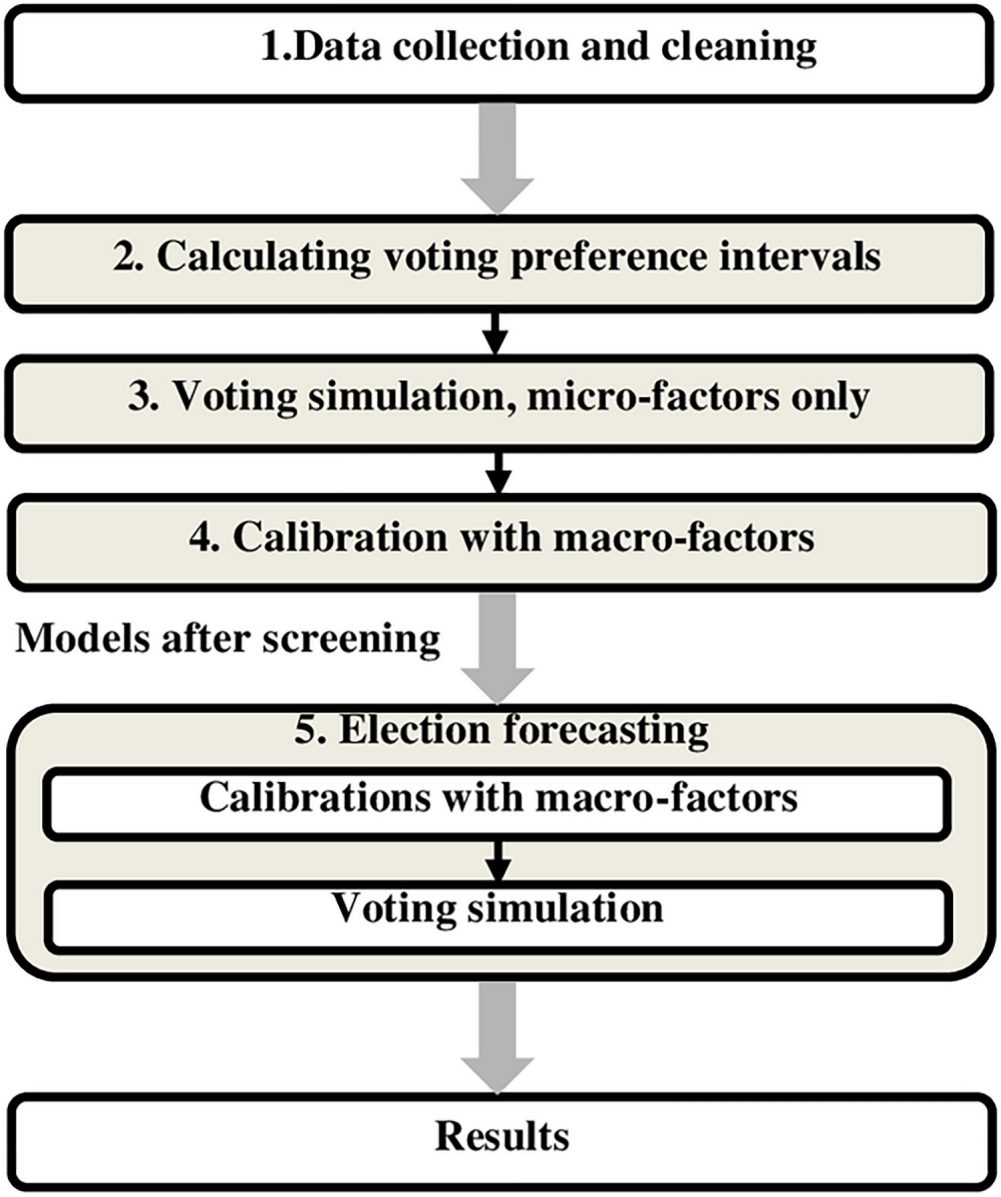
Forecasting elections with ABM simulation.

After setting agents’ attributes and these initial rules, we can then simulate how voters have voted in past elections in our platform. Models that are screened against results from past elections and found to reproduce past electoral outcomes within a small margin of error (±2.5%) are then deployed for forecasting an upcoming election in new forecasting simulations. Altogether, our election forecasting method with ABM simulation typically proceeds in five major steps (Fig 1).

The first step is to collect and clean data. There are two primary sets of data: input data (or predictors) and outcome data. Input data include micro-level social-demographic indicators of voters (e.g., age, gender, education, occupation, ethnicity, religious beliefs), macro-level structural indicators of voting environment (e.g., economic growth rate, Gini coefficient, unemployment rate), candidate features (e.g., incumbency, age, gender, education, scandals), and shock events that may have impacted elections (e.g., 9/11, the 2008 financial crisis). Outcome data are the actual election results in the past. These data will be used for both constructing preliminary models and then screening for robust models that can accurately reproduce historical electoral results.

The second step is to define the initial voting rules for ABM simulation by calculating the "voting preference intervals" of predictive variables. Given that only a limited number of elections are in record (number of observations), regressions with multi-variables are inappropriate. Instead, we derive initial voting rules with simple single-variable regressions (e.g., with share of votes by a party or candidate as the dependent variable, and age as the lone independent variable). This produces a series of results with each of the predictive variables that very roughly suggests how a particular variable may shape the way an agent (i.e., a voter) might have voted or will vote. Since we cannot know exactly the precise effect of a predictive variable upon a voter’s preference, we assign a range rather than an exact value to the effect. We label such a range of voter’s preference as "voting preference interval." Combining all the voting preference intervals of an agent, we obtain an aggregated voting preference interval as the initial rules for guiding an agent’s voting behavior in the ABM simulations.

Briefly, the voting preference interval of a predictive variable is jointly determined by the significance level (P value) and the positive or negative correlation of the variable with the election outcome. The rules for providing initial voting preference intervals are as follows:

$$Voting\;preference\;interval=\left\{\begin{array}{c} \left[2\%,5\%],\;(P\;value\leq 0.15,"+"\right)\\ \left[-5\%,-2\%],\;(P\;value\leq 0.15,"-"\right)\\ \left[0\%,2\%],\;(P\;value>0.15,"+"\right)\\ \left[-2\%,0\%],\;(P\;value>0.15,"-"\right) \end{array}\right\}.$$

More concretely, when the P value of the regression coefficient β is ≤ 0.15 and the variable is positively associated with the election result, the voting preference interval is set to be [2%, 5%]; when the P value of the regression coefficient β is ≤ 0.15, while negatively related to the election result, the voting preference interval is set to be [-5%,-2%]; when the P value of the regression coefficient β is> 0.15, and it is positively associated with the election result, the voting preference interval is [0%, 2%]; when the P value of the regression coefficient β is> 0.15, while negatively related to the election result, the voting preference interval is [-2%, 0%]. Again, these intervals merely serve the purpose of getting the simulation started.

The third step is to simulate voting behavior by voters. We first generate agents in the ABMs according to the known distributions of demographic figures (e.g., in a past election, 49% of the whole voting population was male, whereas the rest was female). We then assign each agent a specific "voting preference interval" based on their unique attributes. Afterwards, we simulate elections by allowing all the agents to vote in elections according to their individual "voting preference interval" in two steps: (1) whether to vote or not, and (2) whom to vote for. Voters with an exact 0% preference (i.e., an agent does not care which side wins in an election) do not vote, while voters with a non-zero preference vote according to their preferences.

The estimated vote shares are obtained by calculating the total votes cast. We run the ABM simulations 100 times and attain final vote shares as the averaged results of these simulations. The estimated vote shares will then be compared to the actual election results in history. Models with large prediction errors (e.g., more than ±10%) will be discarded. In this phase, only models that produce results within the ±10% error margins are retained for the next step.

The fourth step is to incorporate the effects of macro-level socioeconomic factors, candidates’ attributes, and shock events (e.g., 9/11, the 2008 financial crisis) into the simulations. Again, we estimate the effect of each variable on election results based on simple regression analysis. The quantified effects of these variables will then be aggregately incorporated into the prediction process of the fitted models selected in the third step, generating adjusted forecasting results. More specifically, we proceed in two sub-phases: 1) We incorporate the effects of socioeconomic factors into our simulations and require the simulations to reproduce historical election results more accurately, with a narrower range of error less than ±5%. 2) We then incorporate the effects of candidates’ attributes and shock events into the simulations and require the simulations to reproduce historical election results even more accurately, with a much narrower range of error less than ±2.5%. Only models that survive these two rounds of screening are retained for forecasting the upcoming election.

The final step is to forecast an upcoming election. Survived models from the fourth step are employed to simulate and forecast the upcoming election, using real-time and projected data of selected micro-level and macro-level predictive variables for the election year. For some socioeconomic data (e.g., economic growth rate), we rely on the nearest quarterly data that are publicly available ahead of the election. For example, as for the U.S. general election in November 2020, we use data from the second quarter of 2020. For demographic data (e.g., how many voters will be 40–59 years old or more than 60 years old), we extrapolate from existing statistical data. The simulation process closely follows the same steps as described above, again simulating with 100 runs. The final forecasted vote share will be estimated according to the averaged simulation results of all the used models by the 100 runs.

**Fig 2:**
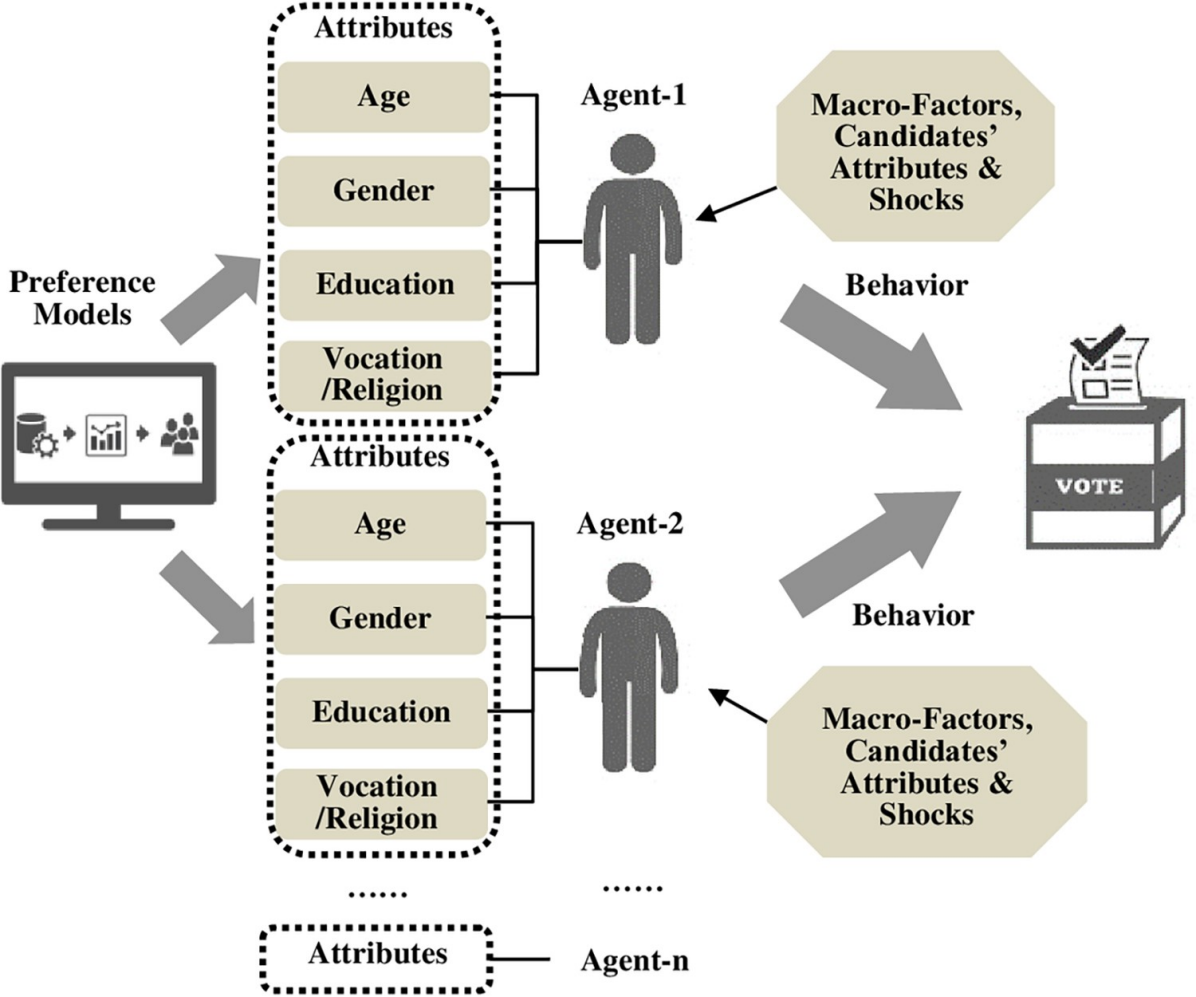
A Schematic illustration of simulating How agents vote with ABM.

These standardized procedures are schematically captured in Fig 2 as below. Four distinct features of our ABM forecasting platform are worth emphasizing. First and foremost, evidently, our ABM-based forecasting does not rely on opinion polls or social media data at all. Our approach thus is distinct from the traditional forecasting techniques and more recent big-data methods. Second, the platform can forecast the share of votes by candidates or parties rather than merely their chance of win. Third, our simulations allow for deriving forecasted results several months or even a year before the real-world elections, securing a longer lead time. Finally, if desired, our simulations help gauge how different blocs of voters with varying attributes have voted in past elections and are likely to vote in a coming election.

## Two live experiments

We now explicate two live experiments we performed in 2020 to show that our election forecasting based on ABM has consistently delivered results with impressive accuracy. All forecasted results have been released online to the public at least two days before the elections, and their online links can be found in the appendixes. In fact, we have obtained our forecasted results about two months ahead of the elections. To avoid affecting the actual electoral results, however, we have deliberately released our results only two days before the elections. For the sake of space, fully detailed descriptions of these experiments are presented in the appendixes. (For our earlier forecasting efforts, which have also been fairly accurate, please visit our official website: www.ccda.fudan.edu.cn).

### Experiment 1: Live forecasting of the 2020 Taiwan general election

At 10 am (Beijing time) on January 9, 2020 (two days ahead of the election date in Taiwan), we released our forecasted results for the 2020 Taiwan general election. This real-time forecasting experiment based on ABM follows the standardized forecasting procedures detailed in the previous section.

For the Taiwan election, we designed two sets of models with different input variables. The key difference between the two sets of models is that group A models contain occupation (agriculture, manufacturing, or service sector), whereas group B models replace it with religious beliefs. These two sets of models were then deployed to forecast the election respectively. The overall results of the two sets of models turned out to be very close. More concretely, the first set of models (the group A models), with 13 individual models, predicted that the Green Camp (the incumbent, Tsai Ing-wen) will receive anywhere between 55.17% (lowest) and 59.48% (highest) of the popular vote, with the averaged share of vote being 56.92%. The second set of models (the group B models), with 611 individual models, predicted that the Green Camp will receive anywhere between 54.06% (lowest) and 59.74% (highest) of the total vote, with the averaged share of vote being 56.26%. [Note: As we recheck the results, we discover that due to an unwarranted error of calculating the means of the results obtained by all the models, our released results (56.86% and 56.42% respectively for group A models and group B models) are slightly different from the correct calculation (56.92% and 56.26% respectively for group A models and group B models). Despite the tiny numerical differences, we sincerely apologize for our mistake.]

Our forecasted results are proven to be extremely close to the final actual election result in 2020. In the actual election, the incumbent Tsai won with 57.13% of the popular vote. In fact, the error difference between our forecasted results and the actual result is only 0.21% for group A models and 0.87% for group B models (see Table 1), both less than 1%. More profoundly, the accuracy of our forecasted results based on ABM simulations greatly outperformed pre-election opinion polls and expert surveys (see the Appendix I for details).

**Table 1:** Forecasted versus actual result in the 2020 Taiwan general election.

	Forecasted Result			Actual Result		Difference	
	Models: Group-A	Models: Group-B	Predicted Winner	Relative Share of Votes	Actual Winner	Models: Group-A	Models: Group-B
**Green Camp**	0.5692 (0.5517, 0.5948)	0.5626 (0.5406, 0.5974)	☆	0.5713	☆	-0.0021	-0.0087
**Blue Camp**	0.4308 (0.4052, 0.4483)	0.4374 (0.4026, 0.4594)		0.4287		0.0021	0.0087

### Experiment 2: Live forecasting of the 2020 U.S. general election

At 12 am (Beijing time) on November 1, 2020 (two days before the presidential election in the United States), we released our computer simulation-based forecasting for the relative shares of votes in the upcoming 59th US presidential election. Due to the budget limit, we can only perform ABM simulation-based forecasting for six selected states (Michigan, Ohio, Pennsylvania, Indiana, Virginia, and Missouri). [Note: In the long run, it will be desirable to be able to forecast the results for all major swing states with ABM simulation. Such an enterprise, however, requires far more resources than what we can afford now.] For these six states, our simulation has produced the forecasted results in early Sept. In fact, we simulated the election results three times, on April 23, July 05, and then finally on September 28 of 2020.

Again, we created two sets of models with different input variables. A key difference between group A models and group B models is that among the variables in our models, the former contains occupation in which sector (agriculture, manufacturing, or others) whereas the latter replaces it with ethnic background. These two sets of models were then deployed to forecast the real-world election respectively. Similar to the results generated in our simulation of the 2020 election in Taiwan, the overall forecasted results for the 2020 US presidential election obtained by the two sets of models are also very close (see Table 2).

**Table 2:** Forecasted versus actual result of the 2020 U.S. presidential election in six states.

State	Candidates	Forecasted Result			Actual Result (updated 2020.11.17 3.40AM) Source: Associated Press	
		Models: Group A	Models: Group B	Forecasted Winner	Relative Share of Votes	Actual Winner
**MI**	Trump-Pence	0.4546 (0.4409, 0.4682)	0.4443 (0.4305, 0.4582)		0.4867 (99%)	
	Biden-Harris	0.5454 (0.5318, 0.5591)	0.5557 (0.5418, 0.5695)	☆	0.5133 (99%)	☆
**OH**	Trump-Pence	0.5075 (0.4952, 0.5198)	0.5089 (0.4966, 0.5212)	☆	0.5414 (95%)	☆
	Biden-Harris	0.4925 (0.4802, 0.5048)	0.4911 (0.4788, 0.5034)		0.4586 (95%)	
**PA**	Trump-Pence	0.4796 (0.4666, 0.4926)	0.4756 (0.4625, 0.4887)		0.4947 (99%)	
	Biden-Harris	0.5204 (0.5074, 0.5334)	0.5244 (0.5113, 0.5375)	☆	0.5053 (99%)	☆
**IN**	Trump-Pence	0.5165 (0.5044, 0.5285)	0.5364 (0.5248, 0.5480)	☆	0.582 (99%)	☆
	Biden-Harris	0.4835 (0.4715, 0.4956)	0.4636 (0.4520, 0.4752)		0.418 (99%)	
**WV**	Trump-Pence	0.6169 (0.6073, 0.6265)	-	☆	0.6981 (99%)	☆
	Biden-Harris	0.3831 (0.3735,0.3927)	-		0.3019 (99%)	
**MO**	Trump-Pence	0.5560 (0.5449, 0.5671)	0.5539 (0.5427, 0.5650)	☆	0.5794 (97%)	☆
	Biden-Harris	0.4440 (0.4329, 0.4551)	0.4461 (0.4350, 0.4573)		0.4206 (97%)	

As shown in Table 2, our forecasting has correctly predicted the outcomes in all six states. In particular, we correctly forecasted that Trump and Pence will win in Ohio but lose in Michigan and Pennsylvania. Unfortunately, our forecasting has also over-estimated the support for Biden and Harris, as many independent or aggregated polls did. However, our forecast has been closer to the actual outcome than most polls (see the appendix II for details). As our first attempt of forecasting the U.S. presidential election, we deem our forecasting effort using ABM to be good despite a larger margin of errors than our earlier efforts of forecasting Taiwan general elections.

## Conclusion

Forecasting elections remains a challenging endeavor. Using ABM simulations, we have performed several experiments of real-time election forecasting since 2015. All of our forecasted results were preregistered and made publicly available several days ahead of the elections. Although we typically obtained our forecasts months before the actual election, we always hold these results in order to avoid being accused of affecting the real world outcome (e.g. [25]). So far, all of our forecasted results have proven to be fairly accurate when compared to the eventual outcome of these elections. Moreover, our forecasted results obtained several months before the election day have consistently performed better than most polls back then. Our experiments therefore point to ABM as a powerful, perhaps even revolutionary, tool for forecasting elections. Compared with traditional forecasting methods, the agent-based simulation approach boasts several remarkable advantages.

First, our ABM platform operates on objective data and uses computational modeling instead of relying on opinion surveys, expert judgments, and fragmented news information. Second, our ABM platform brings together micro-level and macro-level predictive variables. Voters are brought back in as principal decision-making agents in the forecasting process, making our forecasting much closer to the real voting process than opinion aggregations and regression models. Third, election forecasting based on ABM is grounded in well-established electoral studies in political science. Variable selection, data collection, and model construction for the ABM forecasting are closely guided by electoral theories in political science, a process where political scientists work in close collaboration with computer scientists. Doing so generates more accurate results and provides deeper insights into the explanation of the outcomes of interest. Fourth, and closely related to the third, forecasting and explanation mutually benefit each other [26–28]. Electoral forecasting based on ABM simulations rests on robust political science theories, has higher analytic tractability, and enhances our cumulative knowledge about how elections work in different settings. In particular, our ABM platform allows us to use predictive factors varying depending on electoral circumstances. For instance, ethnicity is no doubt an important predictor for US presidential elections and should be considered in any forecasting exercise, while this variable is not that important to simulating Japanese elections. Overall, the novel forecasting approach of ABM simulations enables us to make forecasts on an earlier date with high predictive accuracy and more explanatory power, which shows the most promise for future election forecasting.

Looking ahead, we see several directions for further exploration. First, various machine learning tools can potentially aid the selection of models and equations in the second and third rounds of model screening. Second, forecasting multi-party elections under more complex electoral landscapes is certainly far much challenging, and it will be extremely interesting to test whether ABM-based platform can perform equally well in such settings. Overall, we are confident that through continuous improvement, our ABM-based platform can potentially bring transformational change to election forecasting.

## Supporting information

S1 File Appendix I. Experiment 1: Forecasting the 2020 Taiwan general election.
(DOCX)

S2 File Appendix II. Experiment 2: Forecasting the 2020 U.S. presidential election.
(DOCX)
