# Supplementary material for: Forecasting elections with agent-based modeling: Two live experiments
Source: PLoS One. 2022 Jun 30;17(6):e0270194. doi: 10.1371/journal.pone.0270194 (PMC9246136; doi:10.1371/journal.pone.0270194)
Supplement: S1 File — Experiment 1: Forecasting the 2020 Taiwan general election. (DOCX) [file pone.0270194.s001.docx]

**Appendix I**

**Experiment 1: Forecasting the 2020 Taiwan General Election**

**Experiment Environment**

- Processor: Intel® Xeon® Platinum 8269CY CPU @ 2.50GHz 3.10GHZ, 32 core
- Memory: 64 GB
- Operation system: Windows Server 2019 Datacenter (64-bit)
- Platform: Microsoft Visual Studio 2019, GAMA 1.8.1
- Language: C++, GAML

**Data**

Prior to the forecasting, our team collected data on a host of variables and indicators that cover historical election results, voters’ demography, socio-economic variables, candidate attributes, shock events, and other factors. Our whole dataset contains more than 50 variables, covering the election years of 2000, 2004, 2008, 2012, 2016, and 2019. Data from 2000 to 2016 are used to construct, train and select forecasting models. For the live forecasting experiment of the 2000 election, we rely mostly on data from 2019.

For the list of variables and related sources, see Table A1 below.

**Generating agents with different combinations of attributes**

For Taiwan, our models for ABM simulation consider five micro-level attributes of voters: age (A), gender (G), education (E), occupation (O), and religious belief (R). Due to limitations imposed by computational power, we assign each agent in the ABM flatform four attributes (characteristic features of voters). As a result, two sets of comparable model groups (group A and group B) are formed. The key difference between group A models and group B models is that the former contains occupation (agriculture, manufacturing, or others), whereas the latter replaces it with religious beliefs, as follows.

Group A: $Agent Attributes=Random\left( A_{g},G_{h},E_{i},O_{j} \right)$, $1\leq g,i,j\leq3, 1\leq h\leq2$

Group B: $Agent Attributes=Random\left( A_{g},G_{h},E_{i},R_{k} \right)$,$1\leq g,i,k\leq3, 1\leq h\leq2$

Agents for simulations are generated according to the number of Taiwanese voters and the distributive patterns of voter attributes in each of historical elections. Due to the constraints of computational capacity, we set the representative ratio of the simulated number of agents to the actual number of Taiwanese voters to be 1:10.

The platform then randomly assigns four sub-attributes of age, gender, education, occupation (or religious belief) to each agent. For example: for group A models, an agent can be a female (G_2_) who is 20-40 years old (A_1_), with a high school education (E_2_), and working in the service sector (O_3_). For group B models, an agent can be a male (G_1_) at age of 41-60 (A_2_), with college education or above (E_3_), and going to church (R_2_).

Each attribute has several sub-categories. Specifically, age is divided into three sub- attributes: 20-40 years old (A_1_), 41-60 years old (A_2_), and above 61 years old (A_3_); gender: male (G_1_), female (G_2_); education: middle school and below (E_1_), high school (E_2_), college and above (E_3_); occupation: agriculture (O_1_), manufacturing (O_2_), service sector (O_3_); religious belief: temple (R_1_), church (R_2_), and non-religious (R_3_). Hence, agents in the ABMs are featured with different combinations of the four attributes.

Notably, the distributive patterns of attributes of all modeled agents in the ABMs are kept exactly the same with the attribute distribution of real voters in previous elections.^[[1]](#footnote-1)^ We run 100 times the process of agent generation to ensure that all voter types are covered.

**Voting preference interval**

Voting preference intervals centered upon micro-level predictive variables are derived from regression results using the historical data of previous general elections in Taiwan (2000, 2004, 2008, 2012, and 2016). We first run binary regressions and then obtain the coefficients of each micro-level variable against the actual election results (vote share of the Blue Camp). The voting preference interval of a variable is jointly determined by the significance level (P value) and the positive or negative correlation of the variable with the election outcome. The detailed rules for providing initial voting preference intervals are described in the main body of the article.

These roughly-defined rules, however, do not have to be very accurate in the first place because with guided machine learning, the ABM simulation will automatically select fitted models that can reproduce results that are close to the actual voting results of historical elections. Combining all the voting preference intervals of an agent, we obtain an aggregated voting preference interval as the initial rules for guiding an agent’s voting behavior in the ABM simulations. These initial rules serve the purpose of getting the simulation started.

All the agents make their electoral decision in the platform according to their individual "voting preference interval" in two steps: (1) whether to vote or not, and (2) whom to vote for. Voters with an exact 0% preference (i.e., an agent does not care which side wins in an election) do not vote, while voters with a non-zero preference vote according to their preferences.

**Election simulation**

To simulate voting behavior by voters, we first generate agents in the ABMs according to the known distributions of demographic attributes across the population of eligible voters. With the scale being set at 0.5%, the voting preference interval of each variable, be it [2%, 5%], [0%, 2%], [-2%, 0%], or [-5%, 2%], can further be divided into either 5 or 7 levels.

The estimation of the final vote shares involves compiling the decisions by all agents with different attributes in the ABMs. Therefore, all sub-features of the four voter attributes (11 different sub-features for group A and in group B) need to be considered all together. We treat a set of voting preference values based on the distribution of 11 sub-features as a model. A new model is generated when the voting preference value of a particular attribute moves one scale up or down within the interval while others remain unchanged. As a result, for both group A and group B, the total number of agent-based models formed with varying combinations of the 11 sub-features at the very beginning is 5^10^ × 7 = 68,359,375. We run the simulations with different models 100 times.

**Model selection**

We then select models that can roughly reproduce the actual election results of previous elections within a pre-determined margin of error. Only models that possess a certain level of accuracy will be retained for the next round. The screening process proceeds in two steps.

(1) In the first step, models are simulated with micro-level predictive variables only. The number of historical elections is $n=5$. The result (vote share of the Blue Camp) predicted by each model is $s_{i}$($1\leq i\leq n=5$), and the actual result of a historical election is $r_{i}$($1\leq i\leq n=5$). The prediction error is thus $d_{i}=s_{i}-r_{i}$($1\leq i\leq n=5$). In this phase, the error margin is set to be $\delta=0.1$. Models with prediction error greater than$\delta=0.1$ (i.e., $\left| d_{i} \right|>\delta=0.1$) will be dropped.

After the first round of screening, a total number of 8,736,938 models survives in group A, with the survival rate being 12.78%, while a total number of 7,826,545 models in Group B survived, with the survival rate being 11.45%.

(2) In the second step, we incorporate the effects of socioeconomic factors, candidates’ attributes, and shock events. We obtain the estimated effect of each of these predictive variables on the election result based on simple regression analysis using the data of historical elections. The aggregated effect of all the macro-level variables is set as $m_{i}$($1\leq i\leq5$). We also define a narrower margin of error $\theta=0.05$. The result (vote share of the Blue Camp) predicted by each model is $s_{i}$($1\leq i\leq5$), and the actual result of a historical election is $r_{i}$($1\leq i\leq5$). The prediction error $p_{i}={m_{i}+s}_{i}-r_{i}$($1\leq i\leq5$). Models with the error margin greater than$p_{i}=0.05$ (i.e., $\left| p_{i} \right|>\theta=0.05$) will then be dropped.

After the second round of screening, there were only 13 models surviving in group A (with a survival rate of 0.000149%) and 611 models surviving in group B (with a survival rate of 0.007807%). These are the models we deploy for the real-time forecasting exercise.^[[2]](#footnote-2)^

**Forecasting the 2020 Taiwan general election**

We use the survived models after screening to simulate and forecast the result of the 2020 general election in Taiwan (see Fig. A1). For macro-level social and economic data, we rely on the updated official data available ahead of the election date (i.e., data for the third quarter of 2019). For micro-level demographic data, we extrapolate from existing historical data. The real-time forecasting experiment based on ABM simulations closely follows the standardized forecasting procedures detailed in the main body of the article. Our final simulated results were obtained on Nov. 26, 2019, over a month before the election.

**Fig. A1: The interface of forecasting the 2020 Taiwan general election with ABM** **simulations**


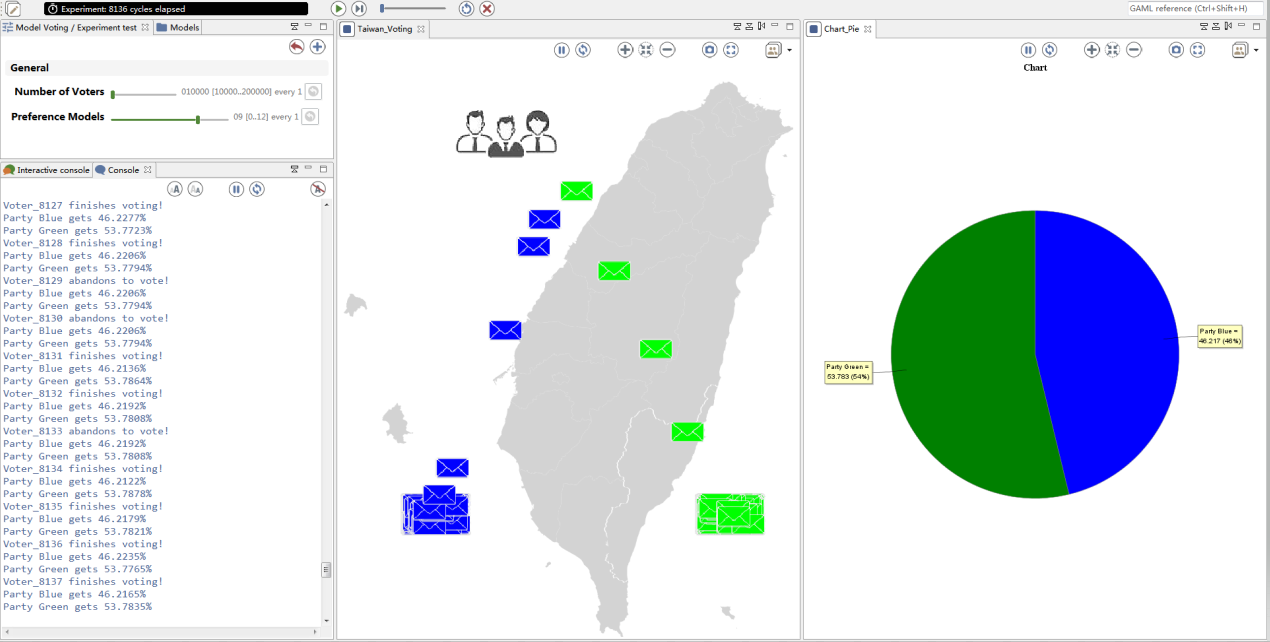


We target the vote share of the Blue Camp as the forecasting goal. The forecasting exercise proceeds in two key steps. First, we simulated the election based on micro-level variables and calculated the votes. As forecasted by models in group A, the Blue Camp will receive anywhere between 49.77% (lowest) and 54.08% (highest) of the total votes. For models in group B, the Blue Camp will receive anywhere between 49.51% (lowest) and 55.19% (highest) of the total votes. The second step was to incorporate the effects of various macro-level variables. The aggregated effect of all the macro-level variables was estimated to be -0.0925.

The final forecasted results were then generated by combining the results obtained by the simulation with micro-level variables with the aggregated effect of all macro-level variables: Group A [0.4052, 0.4483] (lowest, highest); Group B [0.4026, 0.4594] (lowest, highest). Meanwhile, the average forecasted result of the 13 models in group A is $\frac{\sum_{i=1}^{13} a_{i}}{13}=0.4308$. The average forecasted result of the 611 models in group B is $\frac{\sum_{i=1}^{611} b_{i}}{611}=0.4374$.

Eventually, group A models forecast that the Green Camp will receive anywhere between 55.17% (lowest) and 59.48% (highest) of the popular vote. The group B models predict that the Green Camp will receive anywhere between 54.06% (lowest) and 59.74% (highest) of the popular vote. The average forecasted results show that the Green Camp (incumbent, Tsai Ing-wen) will win the election with 56.92% (by models in group A) or 56.26% (by models in group B) of the popular vote.

To avoid being accused of influencing the real-world electoral outcome, we held our forecasted results and released them only two days (10AM, January 9, 2020, Beijing time) ahead of the election date even though we had obtained the results more than one month in advance. (Forecasts Preregistered: <http://www.ccda.fudan.edu.cn/index.php?c=article&id=95>).

On January 11, 2020, Taiwan held its general election. The official result shows that the Blue Camp candidate won 42.87% relative vote, while the incumbent Green Camp won 57.13%. The forecasted vote shares by our platform proved remarkably close to the final real-world election outcome. In fact, the difference between our forecasted results and the actual result is only 0.21% for models in Group A and 0.87 % for models in Group B. Furthermore, our forecasted results show significantly higher accuracy than opinion polls close to the election date (see Fig.A2).

**Fig. A2: The ABM forecasted results and polls prediction results compared**


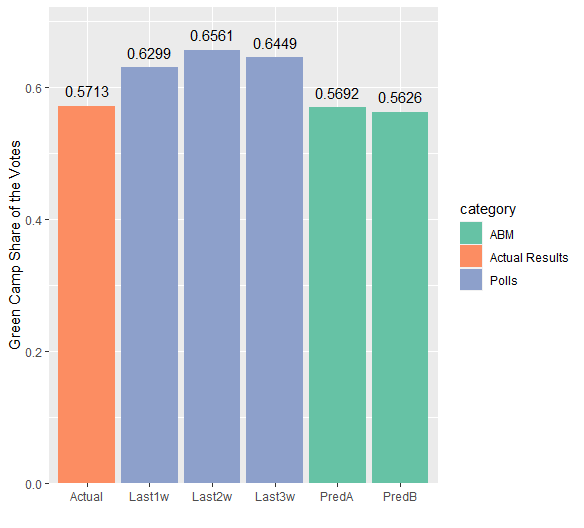


Note: The Actual Result refers to the relative vote share obtained by the green camp vis-à-vis the blue camp in the general election. PredA and PredB are the results respectively forecasted by models in group A and models in group B using ABM simulations. Polls made by various polling houses in Taiwan are available at the "Internet Archive" (https://zh.m.wikipedia.org/wiki/2020年中華民國總統選舉民意調查). We use the polls to calculate average poll results. Among them, Last1w represents a polling average for all polls from December 23, 2019 to January 1, 2020, just one week before the pre-election silence; Last2w represents a polling average for all polls from December 16 to 22, 2019, two weeks before the pre-election silence; Last3w represents a polling average for all polls from December 9 to 15, 2019, three weeks before the pre-election silence.

As shown in Fig.A2, our forecasted results for the 2020 Taiwan general election based on ABM simulations show several clear advantages compared with the average polls results of either three weeks, or two weeks or even one week before the election date. First, the accuracy of ABM forecasts significantly outperforms that of the polls, with its error margin being maintained within 1%. Secondly, the results of the ABM forecasting method can be obtained over half a year in advance if necessary. It therefore allows for longer lead time and provides more policy implications than other methods. Last but not least, ABM simulations consider the effects of various predictive variables at both the macro level and the individual level. Therefore, they present stronger explanatory power and scholarly values for social sciences.

**Table A1: Variables and Data Used for the 2020 Taiwan General Election Forecasting**

**Variables and Data Used in the 1^st^ Round**

**1. Age**

Description of the variable: age group of total citizen population

Category (3): 20-40; 41-60; 61+

Data source: https://statdb.dgbas.gov.tw/pxweb/Dialog/statfile9.asp

**2. Gender**

Description of the variable: gender of total citizen population

Category (2): Male; Female

Data source: https://statdb.dgbas.gov.tw/pxweb/Dialog/statfile9.asp

**3. Education**

Description of the variable: educational background of total citizen population

Category (3): Middle school and below; High school degree or its equivalent; Bachelor's degree or Graduate degree, or their equivalent

Data source: https://statdb.dgbas.gov.tw/pxweb/Dialog/statfile9.asp

**4. Occupation by industry**

Description of the variable: employment status of total citizen population

Category (3): Agriculture; Manufacturing; Others

Data source:  https://statdb.dgbas.gov.tw/pxweb/Dialog/statfile9.asp

**5. Religion**

Description of the variable: religious belief of total citizen population

Category (3): Temple, Church, Religious Unaffiliated

Data source: http://statis.moi.gov.tw/micst/stmain.jsp?sys=100

**Variables and Data Used in the 2^nd^** **Rounds**

**1. Incumbent Status**

Description of the variable: whether a candidate is incumbent.

Binary variable：1; 0

Data source: official record

**2. Voter Turnout Rate**

Description of the variable: total voted divided by total citizen population

Continuous variable

Data source: https://www.cec.gov.tw/

**3. Annual GDP Growth Rate**

Description of the variable: annual GDP growth rate of the year before election years

Continuous variable

Data source: https://www.dgbas.gov.tw/

**4. Unemployment Rate**

Description of the variable: unemployment rate of total citizen population

Continuous variable

Data source: https://statdb.dgbas.gov.tw/pxweb/Dialog/statfile9.asp

**5. Growth Rate of per capita Disposable Income**

Description of the variable: growth rate of per capita disposable income of election years

Continuous variable

Data source: https://www.dgbas.gov.tw/

**6. Low Income Household Rate**

Description of the variable: low income household rate of election years

Continuous variable

Data source: https://www.dgbas.gov.tw/

**7. Gini Coefficient**

Description of the variable: Gini coefficient of election years

Continuous variable

Data source: https://www.dgbas.gov.tw/

**8. Environmental Pollution**

Description of the variable: environmental pollution index

Continuous variable

Data source: https://statdb.dgbas.gov.tw/pxweb/Dialog/statfile9.asp

**9. Candidate Characteristics**

Description of the variable: candidate characteristics index

Continuous variable

Data source: Public Media and Expert Survey

**10. Shock Events**

Description of the variable: shock events that may have impacted elections

Binary variable: 1; 0

Data source: Public Media and Expert Survey

1. For example, if in a historical election, the ratio of male voters versus female voters equals 5.5: 4.5, then the male and female ratio of modeled agents must also meet 5.5: 4.5. The same rule applies to all other attributes. [↑](#footnote-ref-1)
2. To note, according to the standard operational procedures, we need to conduct a third round of screening, requiring the simulations to reproduce historical election with a much narrower range of error less than ±2.5%. However, because survived models in the second round turned out to be very few but perform fairly well, we decide to use these models for live forecasting experiments. [↑](#footnote-ref-2)
