# Supplementary material for: Forecasting elections with agent-based modeling: Two live experiments
Source: PLoS One. 2022 Jun 30;17(6):e0270194. doi: 10.1371/journal.pone.0270194 (PMC9246136; doi:10.1371/journal.pone.0270194)
Supplement: S2 File — Experiment 2: Forecasting the 2020 U.S. presidential election. (DOCX) [file pone.0270194.s002.docx]

**Appendix II**

**Experiment 2: Forecasting the 2020 U.S. Presidential Election**

**Experiment Environment**

- Processor: Intel® Xeon® Platinum 8269CY CPU @ 2.50GHz 3.10GHZ, 32 core
- Memory: 64 GB
- Operation system: Windows Server 2019 Datacenter (64-bit)
- Platform: Microsoft Visual Studio 2019, GAMA 1.8.1
- Language: C++, GAML

**Data**

Prior to the forecasting, our team collected data on a host of variables and indicators that cover historical election results, voters’ demography, socio-economic variables, candidate attributes, shock events, and other factors.

Our whole dataset contains more than 50 variables, covering the election years of 2000, 2004, 2008, 2012, 2016, and 2019. Data from 2000 to 2016 are used to construct, train and select forecasting models. Our simulation also strives to take into account the potential impact of the COVID-19 pandemic. Thus, for our live forecasting experiment of the 2020 U.S. presidential election, we rely mainly on the lastest available data from 2019 and updated data from April, July and September of 2020 respectively as we simulated the election results three times.

For the list of variables and related sources, see Table B1 below.

**Generating agents with different combinations of attributes**

For the United States, our ABM simulation models consider five micro-level attributes of voters: age (A), gender (G), education (E), occupation (O), and ethnicity (T). Each attribute has several sub-categories. Due to limitations imposed by computational power, we assign each agent in our ABM flatform four attributes (characteristic features of voters). As a result, two sets of comparable model groups (group A and group B) are formed. As a result, two sets of comparable model groups (group A and group B) are formed. A key difference between group A models and group B models is that the former contains occupation (agriculture, manufacturing, or others), whereas the latter replaces it with ethnicity, as follows.

Group A: $Agent Attributes=Random\left( A_{g},G_{h},E_{i},O_{j} \right)$, $1\leq g,i,j\leq3, 1\leq h\leq2$

Group B: $Agent Attributes=Random\left( A_{g},G_{h},E_{i},T_{k} \right)$,$1\leq g,i\leq3, 1\leq h\leq2$,$1\leq k\leq4$

Agents for simulations are generated according to the number of American voters and the distributive patterns of voter attributes in each of historical U.S. presidential elections. Due to the constraints of computational capacity, we set the representative ratio of the simulated number of agents to the actual number of American voters to be 1:10.

The platform then randomly assigns four sub-attributes of age, gender, education, occupation (or ethnicity) to each agent. For example: for group A models, an agent can be a female (G_2_) who is 18-44 years old (A_1_), with a college education (E_3_), and working in other sectors (V_3_). For group B models, an agent might be a male (G_1_) at the age above 65 (A_3_), with high school education (E_2_), and white (T_1_).

Each attribute has several sub-categories. Specially, age is divided into three sub-features: 18-44 years old (A_1_), 45-64 years old (A_2_), and above 65 years old (A_3_); gender: male (G_1_), female (G_2_); education: middle school and below (E_1_), high school (E_2_), college and above (E_3_); occupation: agriculture (O_1_), manufacturing (O _2_), others (O _3_); ethnicity: White (T_1_), Black (T_2_), Asian(T_3_), and Latino (T_4_). As a result, agents for the ABM simulations are assigned with different combinations of these sub-features of the four selected attributes.

Importantly, the distributive patterns of attributes of all modeled agents in the ABM simulations are kept the same with the attribute distribution of real American voters in previous elections. For example, in the 2000 presidential election, the male-female ratio of voters in Michigan was 0.4864 versus 0.5136, then the male-female ratio of simulated agents for Michigan in 2000 must also meet the ratio of 0.4864 versus 0.5136. We run 100 times the process of agent generation in order to ensure that all voter types are covered.

**Voting preference interval**

Voting preference intervals centered upon micro-level predictive variables are derived from regression results using the the historical data from previous U.S. presidential elections (2000-2016). We first run binary regressions and then obtain the coefficients of each micro-level variable against the actual election results (vote share of the Democratic Party). The voting preference interval of a variable is jointly determined by the significance level (P value) and the positive or negative correlation of the variable with the election outcome. The detailed rules for providing initial voting preference intervals are described in the main body of the article.

Again, these roughly-defined rules, however, do not have to be very accurate in the first place because with guided machine learning, the ABM simulation will automatically select fitted models that can reproduce results that are close to the actual voting results of historical elections. Combining all the voting preference intervals of an agent, we obtain an aggregated voting preference interval as the initial rules for guiding an agent’s voting behavior in the ABM simulations. These initial rules serve the purpose of getting the simulation started.

All the simulated agents make their electoral decision in the platform according to their individual "voting preference interval" in two steps: (1) whether to vote or not, and (2) whom to vote for. Voters with an exact 0% preference (i.e., an agent does not care which side wins in an election) do not vote. If the aggregated preference turns out to be positive, the agent votes for the Democratic Party, while if negative, the agent votes for the Republic Party.

**Election simulation**

To simulate voting behavior by voters, we first generate agents in the ABMs according to the known distributions of demographic attributes across the population of eligible voters. With the scale being set at 0.5%, the voting preference interval of each variable, be it [2%, 5%], [0%, 2%], [-2%, 0%], or [-5%, 2%], can further be divided into either 5 or 7 levels.

The estimation of the final vote shares involves compiling the decisions by all agents with different attributes in the ABMs. Therefore, all sub-features of the four voter attributes (11 sub-features for group A;12 sub-features for group B) need to be considered all together. We treat a set of voting preference values based on the distribution of 11 or 12 sub-features as a model. A new model is generated when the voting preference value of a particular attribute moves one scale up or down within the interval while others remain unchanged.

Taking Michigan as an example, for Group A , the total number of agent-based models formed with varying combinations of the 11 sub-features at the very beginning is 5^11^=48,828,125 models, while the number of agent-based models formed with varying combinations of the 12 sub-features for Group B is 5^11^×7=341,796,875. We also run the simulations with different models 100 times.

**Model selection**

We select fitted models that can roughly reproduce the actual election results of historical elections within a pre-determined margin of error. Only models that possess a certain level of accuracy will be retained for the next round. The screening process proceeds in two steps.

(1) In the first round, models are simulated with micro-level predictive variables only. The number of historical elections $n=5$. The result (vote share of the Democratic Party) predicted by each model is $r_{i}$($1\leq i\leq n=5$), and the actual result of a historical election is $r_{i}$($1\leq i\leq n=5$). The prediction error is thus $d_{i}=s_{i}-r_{i}$($1\leq i\leq5$). The error margin is defined as $\delta=0.1$. Models with the error margin greater than$\delta=0.1$ (i.e., $\left| d_{i} \right|>\delta=0.1$) will be dropped.

After this round of screening, for Michigan, a total number of 8, 918,090 models survives in group A, with a survival rate of 18.26%, while 48,172,697 models survive in Group B, with a survival rate of 19.73%. For Ohio, a total number of 5,402,417 models survives in group A, with a survival rate of 7.90%, while 24,144,079 models stay in Group B, with a survival rate of 7.06%. For Pennsylvania, a total number of 11,446,607 models remains in group A, with a survival rate of 23.44%, while 15,273,117 models survive in Group B, with a survival rate of 6.26%. For Indiana, 5,796,580 models survive in group A, with a survival rate of 11.87%, while 32,117,304 models survive in Group B, with a survival rate of 13.16%. For Missouri 95,703,125 models survive in group A, with a survival rate of 26.98%, while 8,810,193 models stay in Group B, with a survival rate of 41.44%. For West Virginia, 4,479,567 models survive in group A, with a survival rate of 24.19%. For West Virginia, our platform, however, can create only one set of models (Group A) due to the fact that the state has very low ethnic diversity (i.e., its population are predominantly white), a key modeling varible for Group B.

(2) In the second round, we incorporate the effects of macro-level socioeconomic factors. We obtain the estimated effect of each of these macro-level predictive variables on the election result based on simple regression analysis using the data of historical elections.

The result (vote share of the Democrats) predicted by each model is $s_{i}$($1\leq i\leq5$), and the actual result of a historical election is $r_{i}$($1\leq i\leq5$). The aggregated effect of all socioeconomic variables is set as $m_{i}$($1\leq i\leq5$). For the second round, we also define a narrower margin of error $\theta=0.05$. As a result, the prediction error $p_{i}={m_{i}+s}_{i}-r_{i}$($1\leq i\leq5$). Models with the error margin greater than$p_{i}=0.05$ (i.e., $\left| p_{i} \right|>\theta=0.05$) will then be dropped.

After the second round of screening, for Michigan, a total number of 2,905,535 models survives in group A, with a survival rate of 32.58%, while 17,819,888 models survive in Group B, with a survival rate of 36.99%. For Ohio, a total number of 1,762,565 models survives in group A, with a survival rate of 32.63%, while 7,403,787 models stay in Group B, with a survival rate of 30.67%. For Pennsylvania, a total number of 3,197,649 models survives in group A, with a survival rate of 27.94%, while 3,730,249 models survive in Group B, with a survival rate of 24.42%. For Indiana, a total number of 666,254 models survive in group A, with a survival rate of 11.49%, while 12,738,688 models survive in Group B, with a survival rate of 39.66%. For Missouri, a total number of 11,882,037 models remain in group A, with a survival rate of 12.42%, while 2,907,555 models survive in Group B, with a survival rate of 33.00%. For West Virginia, 2,961,228 models remain in group A, with a survival rate of 66.11%.

(3) In the third round, models are screened by further considering the effects of shock events and candidate attributes (including age, education, face, campaign skills, etc.). An even narrower error margin $\theta$is introduced, but we set $\theta$ differently for the six states. The result (vote share of the Democratic) predicted by each model is $s_{i}$($1\leq i\leq5$) and the actual result of a historical election is $r_{i}$($1\leq i\leq5$). We estimate the effects of shock events and candidate features based on regression analysis using the data of historical elections and evaluation by area experts. The aggregated effect is set as $m_{i}$($1\leq i\leq5$). We define the prediction error $p_{i}={m_{i}+s}_{i}-r_{i}$($1\leq i\leq5$). Models survive the selection process only when $\left| p_{i} \right|\leq\theta$.

After the third round of screening, for Michigan, a total number of 128,724 models survives in group A（$\theta=0.02$）, with a survival rate of 4.43%, while in Group B（$\theta=$0.0115） 39,649 models retain, with a survival rate of 0.22%. For Ohio, a total number of 9,893 models survives in group A（$\theta=0.022$）, with a survival rate of 0.56%, while in Group B（$\theta=$0.02）24,652 models retain, with a survival rate of 0.33%. For Pennsylvania, a total number of 28,798 models survives in group A ($\theta=0.023$), with a survival rate of 0.90%, while in Group B（$\theta=0.022$）12,566 models retain, with a survival rate of 0.34%. For Indiana, a total number of 954 models survives in group A（$\theta=0.03$）, with a survival rate of 0.14%, while in Group B（$\theta=0.025$）20531 models retain, with a survival rate of 0.16%. For Missouri, a total number of 202,192 models survives in group A（$\theta=0.018$）, with a survival rate of 1.70%, while in Group B（$\theta=0.02$）103,726 models retain, with a survival rate of 3.57%. For West Virginia, a total number of 37,569 models survives in group A（$\theta=0.02$）, with a survival rate of 1.27%. These are the survived models we deploy for the real-time forecasting exercise.

**Forecasting the 2020 U.S. presidential election**

We use the survived models after screening to simulate and forecast the result of 2020 U.S. presidential election (see Figure B1).

For macro-level social and economic data, we mainly relied on the updated official data available ahead of the election date (e.g., data for the second quarter of 2020). For micro-level demographic data, we extrapolated from existing historical data. The real-time forecasting experiment based on ABM simulations closely follows the standardized forecasting procedures described in the main body of the article. We performed the live forecasting exercises three times, respectively on April 23, July 05, and then finally on September 28 of 2020, with different updated data available then. We released the final ABM-simulated results (i.e., the results of Septermber 28, 2020) as our forecasted results, while the results of three live forecasting exercises are in fact fairly close.

Again, to avoid being accused of influencing the real-world electoral outcome, we held our forecasted results and released them only two days (12AM, November 1, 2020, Beijing time) before the election even though we had obtained the results months ahead. (Forecasts Preregistered: http://www.ccda.fudan.edu.cn/index.php?c=article&id=107).

**Fig. B1: The interface of forecasting the 2020 U.S. general election with ABM simulations**


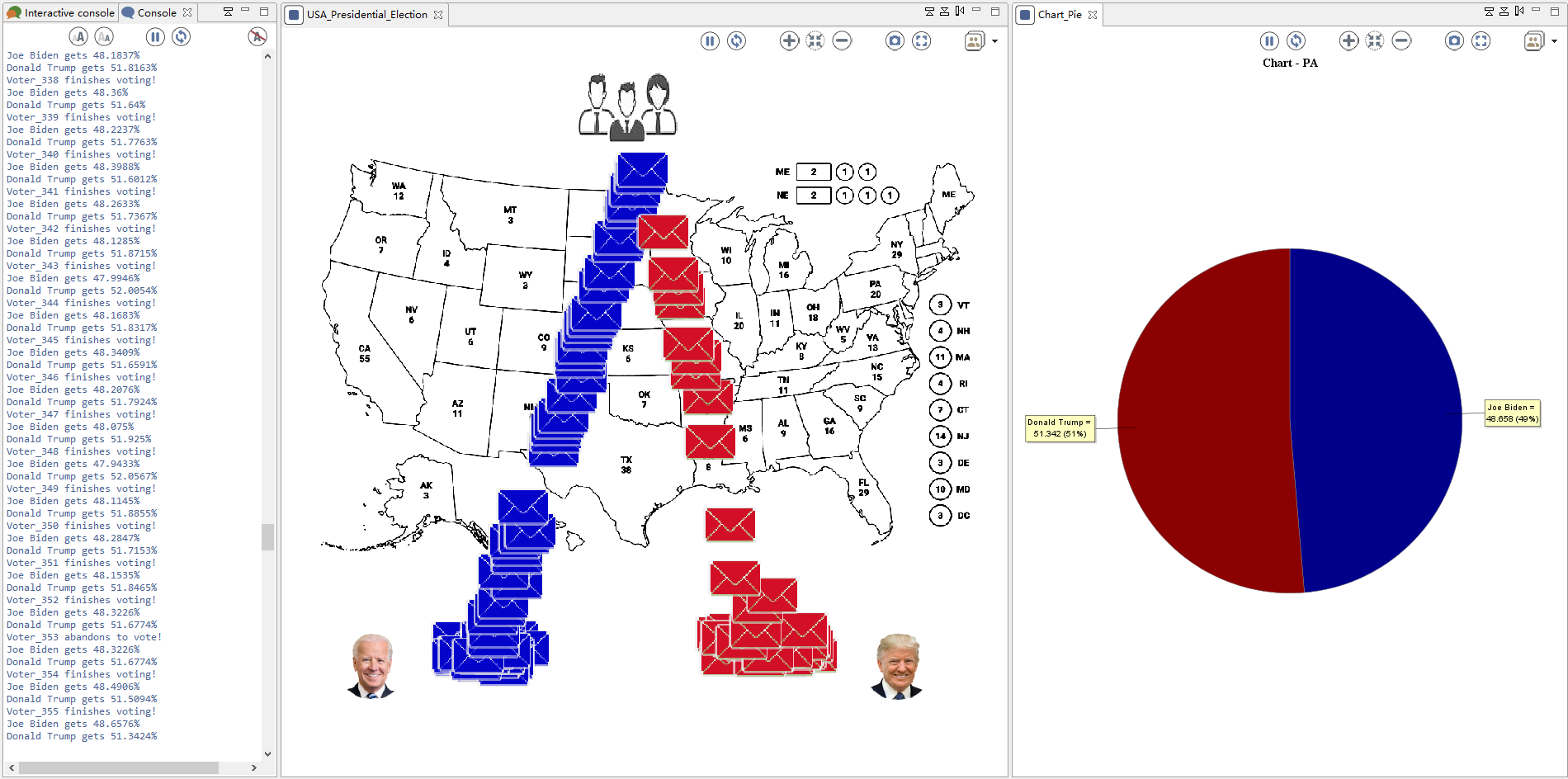


We set the vote share of the Democratic Party as our forecasting goal. The forecasting exercise also proceeds in three key steps. The first step was to simulate the election based on micro-level variables and calculate the preliminary votes. The second step is to adjust the forecasting results by incorporating the effects of macro-level socio-economic variables. The third step is to further adjust the forecasting results by taking into account the effects of candidate attrbutes and shock events. The final forecasted results were then generated by combining the results obtained by the simulation with micro-level variables with the aggregated effect of all macro-level variables, candidate attrbutes and shock events.

For the six states selected for the live forecasting experiment, we predicted which party will win and what will be their vote shares in the 2020 U.S. presidential election. The vote share is estimated by averaging the forecasted results of all simulated models (see Table 2 in the main body of this article).

Taking Michigan as an example, the average forecasted result of the 128724 simulated models in group A is $\frac{\sum_{i=1}^{128724} a_{i}}{128724}=0.5454$(the vote share of the Democratic Party). The average forecasted result of the 39649 models in group B is $\frac{\sum_{i=1}^{39649} b_{i}}{39649}=0.5557$ (the vote share of the Democratic Party). Accordingly, we also estimated the distribution range by setting the error margin as $\pm2.5\%$. Again, taking Michigan as an example, group A models forecast that the Democratic Party will receive anywhere between 53.18% (lowest) and 55.91% (highest) of the total votes, that is, [0.5318, 0.5591]. The group B models forecast that the Democratic Party will receive anywhere between 54.18% (lowest) and 56.95% (highest) of the total votes, that is, [0.5418, 0.5695].

**Fig. B2: The ABM forecasted results and polls prediction results compared**


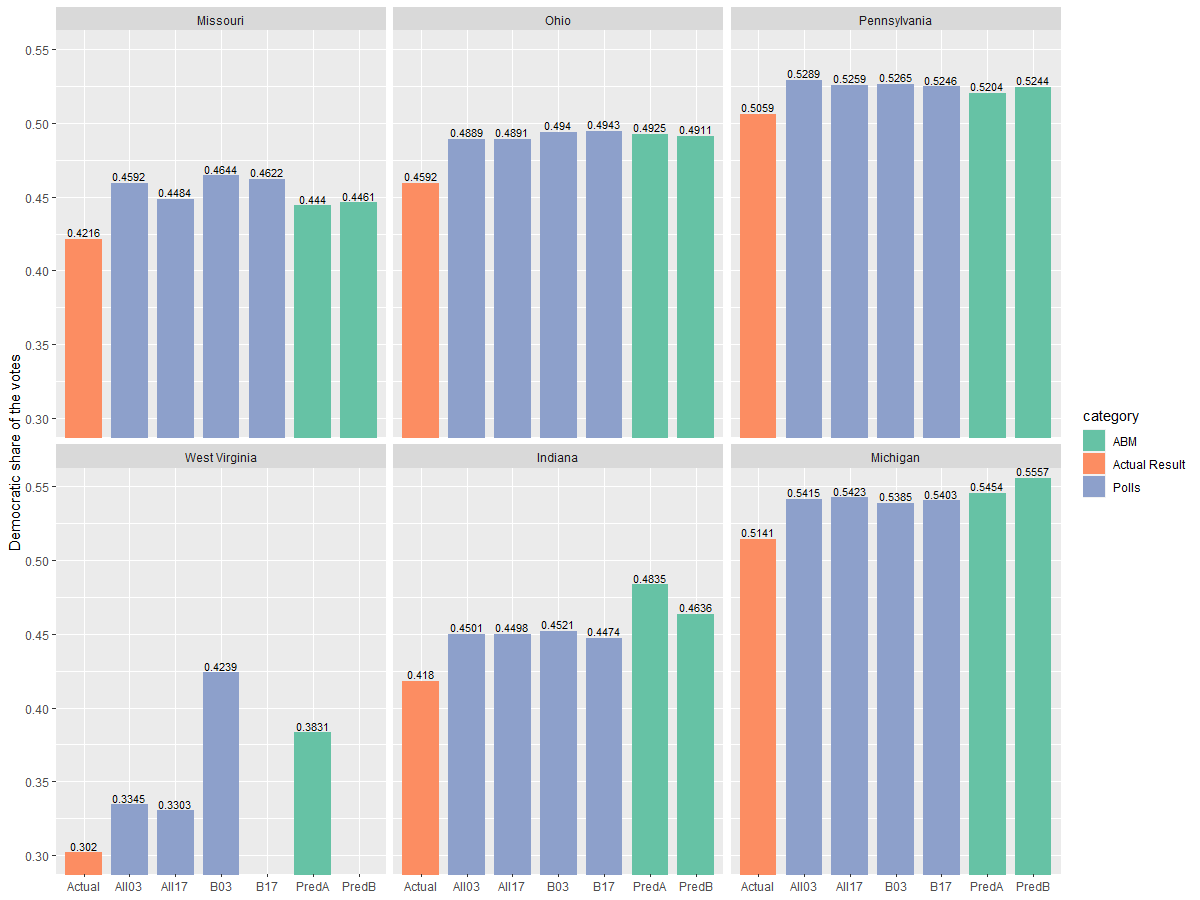


Note: The Actual Result refers to the relative vote share of the Democratic Party vis-à-vis the Republican Party in the 2020 U.S. presidential election. PredA and PredB are the results respectively forecasted by models in group A and models in group B using ABM simulations. The polls data is collected by the "fivethirtyeight" (https://projects.fivethirtyeight.com/2020-election-forecast/). We use these polls to calculate average poll results. All03 represents a polling average using all the polls data available from October 3-31, 2020; All17 represents a polling average using all the polls data available from October 17-31, 2020, which is closer to the election date; B03 and B17 consider only the polls rated B and above.

As shown in Fig. B2, our forecasted results for the 2020 U.S. presidential election based on ABM simulations generally perform better than the average polls prediction results one or half month before the general election. First of all, among the six states forecasted, the accuracy of the ABM forecasting in Missouri, Ohio, and Pennsylvania outperforms that of the polls. Although in Michigan and Indiana, the polls prediction appears closer to the final results of the general election, our forecasts maintain the accuracy in predicting the winner side. In West Virginia, the ABM method performs much better in forecasting the result than B+ level polls. In terms of forecasting accuracy, it is therefore fair to say that the ABM approach is very promising. Second, the results of the ABM forecasting simulations can be obtained more than half a year in advance with lower cost. It therefore offers longer lead time and generates stronger policy values than other methods. Last but not least, ABM simulations consider the effects of various predictive variables at both the macro level and the individual level. Therefore, they present stronger explanatory power and scholarly values for social science research.

**Table B1: Variables and Data Used for the 2020 U.S. Presidential Election Forecasting**

**Variables and Data Used in the 1^st^ Round**

**1. Age**

Description of the variable: age group of total citizen population

Category (3): 18-44; 45-64; 65+

Data source: https://www.census.gov/topics/public-sector/voting/data/tables.html

**2. Gender**

Description of the variable: gender of total citizen population

Category (2): Male; Female

Data source: https://www.census.gov/topics/public-sector/voting/data/tables.html

**3. Educational level**

Description of the variable: educational background of total citizen population

Category (3): Middle school and below; High school degree or its equivalent; Bachelor's degree or Graduate degree, or their equivalent

Data source: https://datacenter.kidscount.org/data/tables/6295-educational-attainment-of-working-age-population-25-to-64

**4. Employment by industry**

Description of the variable: employment status of total citizen population

Category (3): Agriculture; Manufacturing; Others

Data source:  https://apps.bea.gov/iTable/iTable.cfm?reqid=70&step=1&isuri=1#

**5. Ethnicity**

Description of the variable: ethnic background of total citizen population

Category (4): White (T_1_), Black (T_2_), Asian(T_3_), and Latino (T_4_)

Data source: https://www.census.gov/topics/public-sector/voting/data/tables.html

**Variables and Data Used in the 2^nd^ and 3^rd^ Round**

**1. Incumbent Status**

Description of the variable: whether a candidate is incumbent.

Binary variable：1; 0

Data source: official record

**2. Voter Turnout Rate**

Description of the variable: total voted divided by total citizen population

Continuous variable

Data source: https://www.census.gov/topics/public-sector/voting/data/tables.html

**3. Annual GDP Growth Rate**

Description of the variable: annual GDP growth rate of the year before election years

Continuous variable

Data source: https://apps.bea.gov/regional/downloadzip.cfm

**4. Unemployment Rate**

Description of the variable: unemployment rate of total citizen population

Continuous variable

Data source:  https://www.bls.gov/bls/news-release/home.htm#SRGUNE

**5. Growth Rate of per Capita Disposable Income**

Description of the variable: growth rate of per capita disposable income of election years

Continuous variable

Data source: https://apps.bea.gov/regional/downloadzip.cfm

**6. Candidate Characteristics**

Description of the variable: candidate characteristics index

Continuous variable

Data source: Public Media and Expert Survey

**7. Shock Events**

Description of the variable: shock events that may have impacted elections (e.g., 9/11, the 2008 Financial Crisis, the Covid-19 pandemic and candidate scandals)

Binary variable: 1; 0

Data source: Public Media and Expert Survey
